# Physicomechanical Performance and Aging Behavior of Polycarbodiimide-Cross-Linked Accelerator-Free Carboxylated Nitrile Butadiene Rubber Latex Gloves

**DOI:** 10.1021/acsomega.6c02669

**Published:** 2026-05-25

**Authors:** Nadia Wan Azman, Ryota Kamei, Toshikazu Matsuoka, Keisuke Ikeda, Yugo Kubono, Azura A. Rashid

**Affiliations:** a School of Materials and Mineral Resources Engineering, 65270 Universiti Sains Malaysia, Engineering Campus, Nibong Tebal, Pulau Pinang 14300, Malaysia; b 643321 Personal Protective Equipment Technology Dept. Midori Anzen Co., LTD., 5-27-1 Inari, Souka, Saitama 340-0003, Japan

## Abstract

Accelerator-free (AF) carboxylated nitrile butadiene rubber (XNBR) glove systems have been proposed as safer alternatives to conventional accelerator-based formulations; however, prior studies are largely restricted to isolated latex grades or single curing conditions, limiting confidence in industrial robustness and scalability. This study presents the extended systematic evaluation of polycarbodiimide/zinc oxide (PCDI/ZnO)-cross-linked AF-XNBR gloves across key manufacturing variables governing real-world performance. Gloves of three controlled thicknesses (55, 70, and 90 μm), produced from latex grades with varying acrylonitrile contents, are cured over a wide temperature range (65, 90, and 110 °C) to elucidate structure–processing–performance relationships. Mechanical integrity is comprehensively assessed according to ASTM D6319 requirements. Notably, the results demonstrate that AF-XNBR gloves can achieve compliant performance even under low-temperature curing conditions, challenging the prevailing assumption that high thermal input is essential. These findings establish the practical sufficiency and industrial viability of PCDI/ZnO-based AF systems, providing a robust framework for optimization and large-scale adoption.

## Graphical abstract

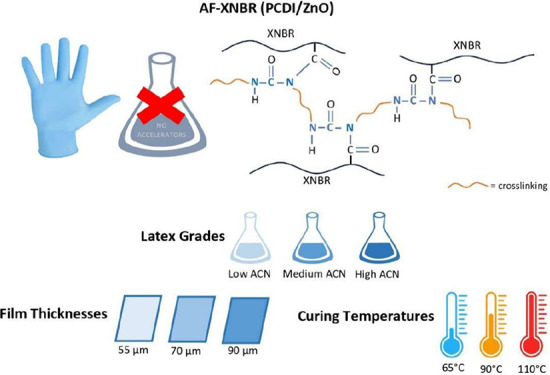

## 1 Highlights

- AF gloves were assessed across latex grades, thicknesses, and temperatures.
- PCDI/ZnO enhances the AF glove performance in strength and permeation.
- AF gloves maintain durability under various temperature conditions.
- Latex grade and ACN content impact the AF glove performance.
- Low-temperature curing produces strong, sustainable AF gloves that meet SDGs.

## 2 Introduction

Accelerator-free (AF) carboxylated nitrile butadiene rubber (XNBR) gloves are a type of nitrile glove that eliminates the use of traditional chemical accelerators such as thiurams, carbamates, or mercaptobenzothiazoles (MBTs), which are commonly used in the vulcanization (cross-linking) of rubber. These gloves are designed to meet growing demands for safer, hypoallergenic alternatives without sacrificing performance.^1^ In conventional nitrile butadiene rubber (NBR) gloves, accelerators are used during vulcanization to improve cross-linking speed and efficiency. However, these accelerators can leave residual chemicals in the gloves, which have been linked to type IV (delayed) allergic reactions in some users.^2^ AF gloves replace those accelerators with nonallergenic cross-linking agents such as carbodiimide functional groups (PCDI), metal oxides (e.g., zinc oxide, (ZnO)), alternative peroxide, or ionic curing agents. In AF formulations, curing is typically done using, for example, PCDI, which reacts with carboxylic acid groups in XNBR to form stable urethane-like linkages, and metal oxide cross-linking, which forms ionic cross-links between XNBR chains and low-temperature curing processes, which conserve energy and reduce thermal degradation. These methods create a flexible but durable polymer network, closely mimicking the properties of sulfur-cured gloves.^3^

The existence of different XNBR grades such as K834, NL129, and NL117 is primarily due to variations in the acrylonitrile (ACN) content and the carboxylic acid monomer level, which directly affect the chemical, physical, and mechanical properties of the latex. ACN is a polar monomer; hence, increasing its content improves intermolecular polarity, leading to tighter molecular packing, reduced gas permeability, and increased resistance to oils, fuels, and solvents. However, it also reduces chain mobility, which can decrease softness and elongation. XNBR grades differ ACN content as shown in Table 1: carboxylic group concentration, molecular weight, particle size, latex viscosity, and stability.^4^ These variations allow manufacturers to tailor the material for specific performance requirements (e.g., chemical resistance, softness, and elongation).^5^

**Table 1:** General Effect of Acrylonitrile (ACN) Content on XNBR Properties Based on Literature Reports^a^

ACN content	characteristics	use cases
low (18–25%)	higher flexibility and softness	gloves requiring comfort and stretch, like examination or medical gloves
	lower oil and chemical resistance	
	better elongation at break	
high (35–45%)	superior oil, fuel, and chemical resistance	industrial gloves for chemical handling, automotive, or oil and gas applications
	lower elasticity	
	stiffer feel	

a Source: Summarized from literature on nitrile rubber structure–property relationships.^5^

The thickness of the gloves plays a crucial role in determining their mechanical strength and flexibility. Thicker gloves generally offer enhanced protection and durability, making them suitable for tasks requiring resistance to punctures, abrasions, and chemical exposure. However, increased thickness can compromise flexibility, dexterity, and tactile sensitivity, potentially leading to reduced hand performance and increased fatigue during prolonged use. Conversely, thinner gloves provide better flexibility and tactile feedback, but they may offer less protection. Therefore, selecting the appropriate glove thickness involves balancing protection requirements with the need for manual dexterity and comfort.^6^ Several studies have explored the impact of the glove thickness on hand performance and fatigue. For instance, research has shown that increasing glove thickness can lead to decreased grip strength and manual dexterity as well as increased muscle activity and fatigue during tasks requiring fine motor skills. These findings underscore the importance of considering glove thickness in the design and selection of gloves for various occupational and clinical applications.^7^

In the manufacture of NBR gloves, the curing temperature is a pivotal parameter that significantly influences the mechanical strength and flexibility of the final product. Curing or vulcanization involves cross-linking polymer chains to enhance the material’s elasticity, durability, and resistance to various stresses. The temperature at which this process occurs dictates the extent and efficiency of cross-linking, thereby affecting the glove’s performance characteristics. Research indicates that optimal curing temperatures lead to improved tensile strength and elongation at break, which are essential for gloves’ protective and functional qualities. For instance, a study on the effect of the curing temperature on NBR revealed that increasing the curing temperature up to a certain point enhances the material’s mechanical properties, but excessive temperatures may lead to degradation. Additionally, the curing process’s thermal parameters influence the glove’s resistance to environmental factors such as temperature fluctuations and chemical exposure. Properly cured gloves exhibit better stability and longevity under varying conditions, which are crucial for applications in medical, industrial, and laboratory settings. Hence, controlling the curing temperature is essential in the production of NBR gloves as it directly affects their mechanical integrity and functional performance. Quantifying and optimizing this parameter can lead to the development of gloves with superior qualities, meeting the stringent demands of various professional environments.^8^

Recent studies have highlighted polycarbodiimide (PCDI) as a promising accelerator-free cross-linking agent capable of reacting with carboxyl groups in XNBR to form stable urethane linkages. In our previous work,^9^ we demonstrated the fundamental feasibility of PCDI for AF-XNBR systems. That study established the mechanism of PCDI–carboxyl reactivity, its ability to generate strong cross-linked networks, and its suitability for low-temperature processing, which addresses important limitations associated with traditional accelerator-based curing. While these findings showcased the chemical strength and low-temperature curing capability of PCDI, several practical and application-centered questions remain unanswered. In particular, glove performance in industrial settings depends heavily on processing parameters such as film thickness, curing temperature, and latex grade, especially the ACN content that contributes to barrier performance and chemical resistance. These parameters were not systematically explored in our earlier work, leaving a significant knowledge gap regarding the robustness and manufacturability of PCDI-based AF-XNBR gloves. In particular, the latest studies^10^ on NBR and related elastomer systems have employed a combination of mechanical testing, spectroscopic analysis, and microstructural characterization methods to better understand cross-linking behavior and material performance. These approaches complement conventional mechanical tests such as tensile strength, modulus, hardness, and swelling analysis, which remain widely used for evaluating elastomer network properties.

In practical production, variations in latex grade, film thickness, and curing temperature can significantly influence cross-linking efficiency, network structure, and ultimately the mechanical and barrier performance of the gloves. Despite their importance, the combined effects of these parameters in PCDI/ZnO-cross-linked AF-XNBR systems remain insufficiently understood. Therefore, this study aims to provide a systematic evaluation of these factors, with particular emphasis on (i) latex-grade-dependent behavior, (ii) thickness-related curing characteristics, and (iii) temperature–structure–property relationships. This work seeks to bridge the gap between laboratory-scale formulation studies and real production conditions. Consequently, this study will show a systematic evaluation of PCDI/ZnO-cross-linked AF-XNBR gloves that is conducted across key manufacturing variables. Gloves of three controlled thicknesses (55, 70, and 90 μm), prepared from latex grades with varying ACN contents, are cured over a wide temperature range (65, 90, and 110 °C) to elucidate structure–processing–performance relationships. This study extends previous findings by systematically addressing key processing variables under realistic conditions, offering new insight into the practical implementation of PCDI/ZnO-based AF systems.

## 3 Experimental Section

### 3.1 Materials

The XNBR latexes K834 (∼27% ACN content), NL129 (∼20% ACN content), and NL117 (∼45% ACN content) were supplied by Kumho Petrochemical Co., Ltd. (Seoul, Korea) and LG Chem (Seoul, Korea). Farben Technique (M) (Penang, Malaysia) provided the latex compounding materials in dispersion form that contained zinc oxide (ZnO) with an average particle size of 0.5026 μm, titanium dioxide (TiO2) with an average particle size of 0.614 μm, and antioxidant. R&M Chemicals Sdn. Bhd. (Selangor, Malaysia) supplied the potassium hydroxide (KOH) as a pH stabilizer, calcium nitrate tetrahydrate [Ca­(NO_3_)_2_·4H_2_O], sodium hydroxide (NaOH), sodium chloride (NaCl), ammonium chloride (NH4Cl), lactic acid, and acetic acid. A coagulant chemical ingredient obtained from Bio Cosmic Sdn. Bhd. (Penang, Malaysia) contained antitack. The supplier of PCDI cross-linkers was Nisshinbo Chemical Inc. (Tokyo, Japan).

### 3.2 Preparation of the Coagulant and Three Types of XNBR Latex Compound

A 12 wt % aqueous solution of calcium nitrate and 1 wt % antitack dispersion were combined to create the coagulant chemical for 55 μm thick nitrile rubber films. The thickness of different films was prepared by adjusting these formulations. Table 2 lists the components of latex compound’s composition. A 5% KOH aqueous solution was used to bring the compound’s pH down to roughly 10. Before the glove dipping process, the latex compound was continuously stirred for at least 16 h for the maturation process.

**Table 2:** Formulation of XNBR Cross-Link System^a^

component	45% XNBR	5% KOH	50% ZnO	50% antioxidant	70% TiO_2_	40% PCDI
phr	100	2	1	0.2	2	0.5

a phr indicates parts per hundred parts of rubber.

### 3.3 Preparation of XNBR Latex Gloves

A KUKA robotic arm equipped with a ceramic hand former was used to carry out the dipping process. The ceramic former was first cleaned, dried, and then dipped into a 12 wt % calcium nitrate coagulant solution at 50 °C for 3 s. It was then allowed for dipping, dwelled, and withdrawn each for 5 s, followed by a 1 min curing process at 115 °C. Using the same dipping parameters, the former was next immersed in the XNBR latex compound. After dipping, it underwent pregelling for 2 min at 50 °C and was then cured for 15 min at the temperatures specified in Table 3. After curing, the glove underwent a postleaching treatment in water at 50 °C for 1.5 min, followed by a 10 s neutralization and a 20 s chlorination process. The glove was carefully stripped from the former and air-dried at room temperature for 10 min. For aging analysis, the gloves were conditioned at 70 °C for 7 days, in accordance with ASTM D573, 2016, to assess their thermal stability.

**Table 3:** Experimental Designs

latex grade	K834	K834	K834	NL129	NL129	NL129	NL117	NL117	NL117
curing temperature (°C)	65	90	110	65	90	110	65	90	110

### 3.4 Fourier Transform Infrared Analysis

Fourier transform infrared (FTIR) analysis was performed using a PerkinElmer Spectrum One spectrometer (Shelton, USA) over a wavenumber range of 550–4000 cm^–1^, with a resolution of 0.5 cm^–1^. Spectral analysis focused on characteristic peak intensities and areas corresponding to specific functional groups present in the samples.

### 3.5 Swelling Test

According to ASTM D471-16a, 2021, the swelling test was carried out using a test piece that was 25 × 50 × 2.0 ± 0.1 mm and was cut from XNBR latex films that had been unaged and aged at 70 °C for 7 days. The sample was then allowed to soak at room temperature for 24 h in toluene. After being cleaned with tissue paper, the swollen sample was weighed. After that, the sample was dried for 30 min at 70 °C in an oven until its weight remained constant. The swelling index (eq 1), in which the weight of the dry sample (g) is denoted by *W*_o_ and the weight of the swelled sample (g) by *W*_s_.

$$\Delta W\left(\%\right)=\left(W_{s}-\frac{W_{o}}{W_{o}}\right)\cdot 100$$ 1

After swelling measurements, the samples were dried in an oven until a constant weight was achieved. The final dry weight was recorded and used to calculate the volume fraction of the XNBR latex samples. The volume fraction of rubber in the swollen network, *V*_r_, was determined using eq 2, where *W*_b_ is the initial weight before toluene immersion, *W*_a_ is the weight after immersion, ρ_r_ is the density of the sample, and ρ_s_ is the density of toluene (0.862 g/cm^3^).

$$V_{r}=\frac{\frac{W_{b}}{\rho_{r}}}{\frac{W_{b}}{\rho_{r}}}+\left(W_{a}-\frac{W_{b}}{\rho_{s}}\right)$$ 2

The cross-link density was calculated using the Flory–Rehner equation (eq 3), where *V*_r_ is the volume fraction of latex in the swollen sample, *V*_s_ is the molar volume of toluene (105.91 cm^3^/mol), and χ is the Flory–Huggins polymer–solvent interaction parameter, taken as 0.39.

$$M_{c}\left(\mathrm{mol}/{\mathrm{cm}}^{³}\right)=-\frac{\left[\ln\left(1-V_{r}\right)+V_{r}+\chi^{2}\right]}{V_{s}\left(V_{r}\exp^{1/3}-\frac{V_{r}}{2}\right)}$$ 3

### 3.6 Durability Property Test

In accordance with JIS K6251, 2017, two distinct specimen shapes were cut from the palm and index finger crotches of the XNBR latex films. These specimen shapes were then cut into dumbbell-shaped type 1. One liter of deionized water is used to create an artificial sweat solution. The test pieces were submerged in water, 20 g of sodium chloride, 17.05 g of lactic acid, 5.01 g of acetic acid, 17.5 g of ammonium chloride, and 2 N sodium hydroxide solution. Attached at a specific location as shown in Figure 1, a test sample was maintained in its typical state of 147 mm for 11 s and then stretched to 195 mm in length before returning to its initial shape for 1.5 s. Each cycle consisted of a complete set of the testing. The action of stretching and then returning to the original length is generally called a loading–unloading cycle or a cyclic deformation cycle. These cycles were repeated continuously until the XNBR latex films failed or ruptured. The point at which the film ruptured was recorded as the durability time. According to an internal standard established by Midori Anzen Co., Ltd., the durability time must be at least 42 min. The durability retention properties are calculated to show how much of the crotch durability is maintained after aging.^11^

retention=(aged/unaged)×100% 4

**Figure 1:**
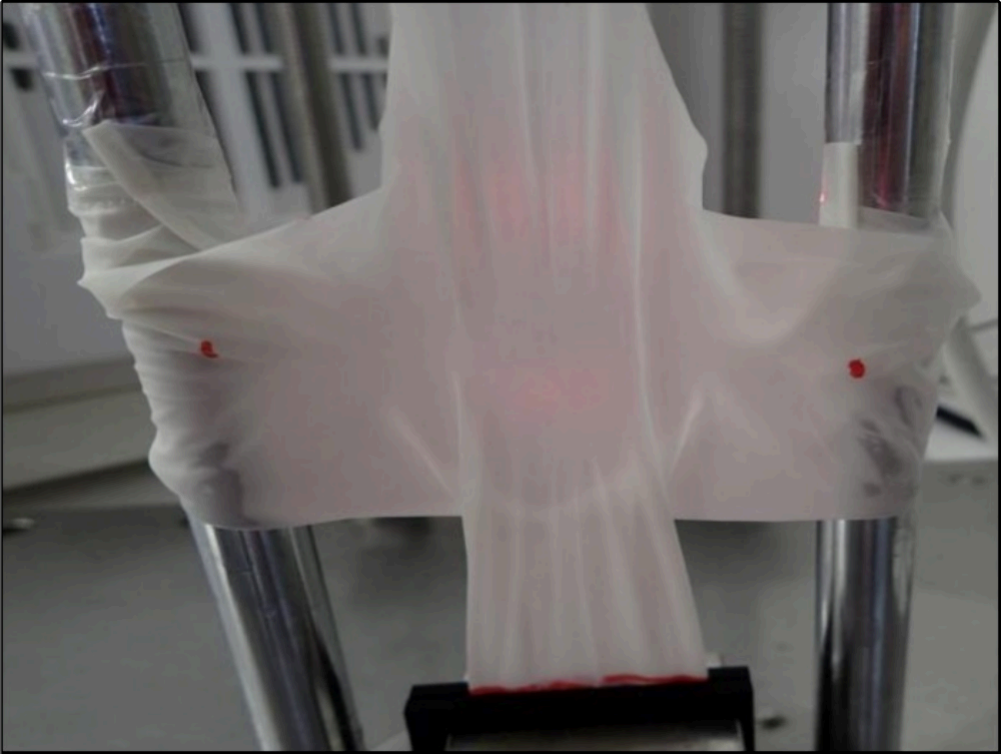
Attachment of sample on the durability test machine.

### 3.7 Tensile Properties and Tear Strength Test

Tensile and tear strength tests were performed on the unaged and aged (70 °C for 7 days) films. ASTM D412-06a, 2021 was followed when performing the tensile test. The purpose of the test was to ascertain whether XNBR latex films could withstand deformation when stretched. Tensile strength, elongation at break, and modulus at 100%, 300%, and 500% are among the results of the tensile test. Five test samples were made in the shape of dumbbells. A tensometer Universal Testing Machine (Model Gotech AI-3000, Taiwan) was used to do the measurement at a speed of 500 ± 50 mm/min, and data was recorded. Test samples for the tear test were cut into crescent shapes (die Type C), in accordance with ASTMD624-00, 2020. After preparing five samples, the average values were determined. A tensometer Universal Testing Machine (Model Gotech AI-3000, Taiwan) was used to measure the tear characteristics at a speed of 500 ± 50 mm/min. The standard temperature and humidity conditions were followed during the testing, which were 23 ± 2 °C and 50 ± 5%, respectively. The mechanical properties of the gloves were evaluated in accordance with ASTM D6319-19, 2023, which specifies a minimum tensile strength of 14 MPa and a minimum elongation at break of 500% for nitrile examination gloves.

## 4 Results and Discussion

### 4.1 Effect of Latex Grade on AF-XNBR Glove Properties

#### 4.1.1 Fourier Transform Infrared Analysis

FTIR spectra of XNBR latex films cross-linked with PCDI for different latex grades (K834, NL117, and NL129) are presented in Figure 2. All samples exhibit similar spectral features, indicating that the overall chemical structure is largely consistent across the different latex grades. Characteristic absorption bands of XNBR are observed, including the nitrile (−C≡N) stretching at ∼2230–2240 cm^–1^ and aliphatic C–H stretching in a region of ∼2850–2950 cm^–1^. The presence of carboxylic acid groups is typically associated with broad O–H stretching (∼2500–3300 cm^–1^) and C=O stretching around ∼1700–1725 cm^–1^.^12^ In the present spectra, these bands are retained with no substantial shifts or intensity changes across all samples. For the PCDI cross-linking reaction, the formation of urea/urethane-like linkages is expected through the reaction between carbodiimide groups and carboxylic acid functionalities. This would typically result in the appearance of characteristic amide/urea carbonyl bands (∼1630–1680 cm^–1^) and possible N–H stretching vibrations (∼3300–3500 cm^–1^), along with a reduction in carbodiimide (−N=C=N−) absorption (∼2100–2140 cm^–1^).^5^

**Figure 2:**
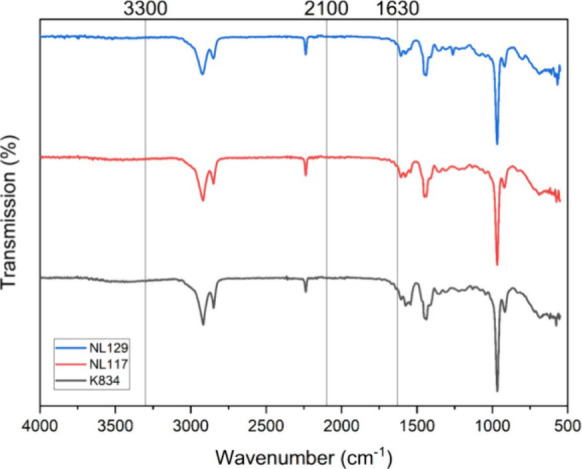
Variation of infrared spectra of XNBR-PCDI film with different latex grades.

However, in the present study, these changes were not distinctly resolved in the spectra. The absence of clearly distinguishable new peaks or significant intensity variations may be attributed to several factors. First, the concentration of PCDI relative to that of the XNBR matrix is relatively low, leading to weak signal contributions from newly formed linkages. Second, overlapping bands from the polymer backbone and existing functional groups may mask the subtle spectral changes. Third, the similar formulation and cross-linking chemistry across all latex grades result in minimal variation between samples.^13^ Therefore, while FTIR provides qualitative confirmation that no major chemical differences exist among the samples, it offers limited sensitivity in conclusively verifying the extent of carbodiimide consumption and urea/urethane formation in this system. These observations are consistent with the swelling and cross-link density results, which also indicate comparable network structures across all samples.^14^

DSC and DMA were not included in this study due to instrument unavailability during the experimental period. While these techniques could provide additional insight into glass transition behavior and cross-link density, their sensitivity may be limited in highly cross-linked XNBR systems with similar formulations, where differences are often subtle. Therefore, the absence of DSC/DMA data does not affect the overall trends observed, but it represents a limitation of the current work. Future studies will incorporate these analyses for a more comprehensive characterization.

#### 4.1.2 Swelling Index Properties

The effect of latex grade was studied using a constant glove thickness of 55 μm in order to minimize the variables. This thickness was chosen to reflect current industry demand for thinner gloves. Based on the results obtained, the swelling index of aged samples is consistently lower than that of unaged samples across all latex grades and curing temperatures. This reduction was found to be statistically significant (*p* < 0.05), signifying that aging leads to a measurable decrease in solvent uptake. Figure 3 shows that XNBR latex gloves produced with latex NL117 had the lowest swelling index, indicating limited toluene penetration during the swelling test. A particular XNBR formulation called latex NL117 was created to maximize chemical resistance. An optimal cross-linking density is probably incorporated into the NL117 formulation to produce a tighter polymer network, which limits solvent molecule penetration to lessen swelling and enhances barrier qualities.^15^ In contrast, the swelling index of all samples decreased after the aging treatment. In particular, the samples with a curing temperature of 65 °C showed notable declines.

**Figure 3:**
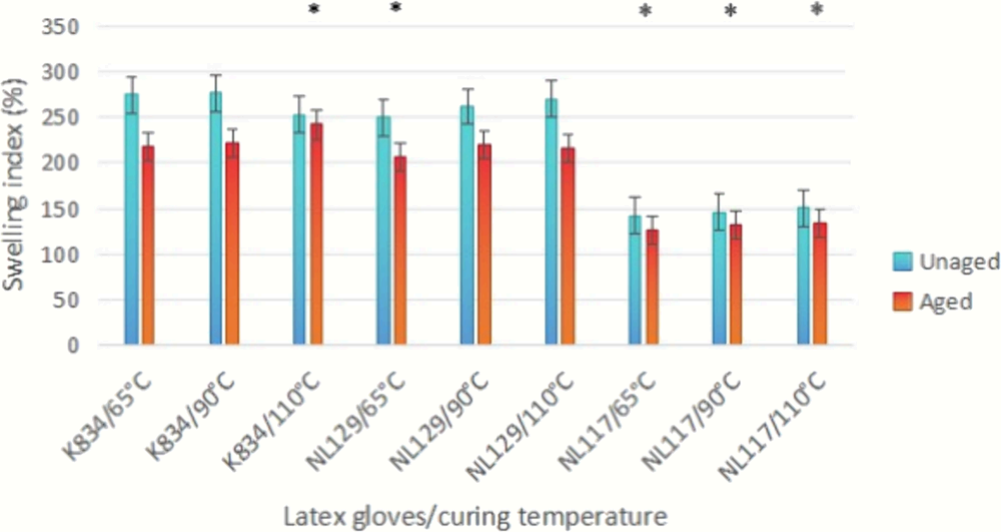
Toluene swelling index of unaged and aged XNBR gloves (thickness of gloves: 55 μm) *p < 0.05.

XNBR gloves with latex K834 and NL129 for all three curing conditions show high swelling (∼250–275%) when unaged. After aging, swelling decreases moderately (∼210–230%), indicating some improvement in solvent resistance, which likely due to additional cross-linking over time. On the other hand, XNBR gloves using latex NL117 show much lower initial swelling (∼140–150%) compared to other latexes throughout all curing conditions. Slight further reduction after aging (∼125–135%), indicating higher inherent solvent resistance even before aging. All samples show reduced swelling after aging, suggesting increased cross-link density over time, which limits solvent penetration. NL117 latex shows superior performance in resisting toluene swelling, both before and after aging. Aging improves resistance across all latex types, though the curing temperature varies. This suggests that cross-linking system effectiveness and curing conditions significantly influence chemical durability.^16^

The swelling index of XNBR gloves reflects how much the polymer absorbs solvents, typically nonpolar ones like toluene or oils. This property is strongly influenced by the polarity of the rubber, which in turn governed by the ACN content. NL117 likely contains a high-ACN content (∼35–45%), making the polymer more polar. Nonpolar solvents do not interact well with polar polymers, which contributes to less solvent penetration. Therefore, gloves made from NL117 absorb less solvent, leading to a lower swelling index, both unaged and aged. Consequently, high ACN increases chemical resistance, particularly against nonpolar solvents, leading to minimal swelling. On the other hand, K834 and NL129 likely contain lower ACN content (∼18–27%), which means that the polymer is less polar. This increases compatibility with nonpolar solvents, so gloves absorb more solvents, which exhibit higher swelling index. After aging, some postcuring or thermal-induced cross-linking occurs, reducing chain mobility and decreasing swelling slightly. On that account, lower ACN provides higher initial swelling, but aging improves solvent resistance by increasing cross-link density.^5,9^

At lower curing temperatures (65 and 90 °C), the slightly higher swelling observed for K834 (27% ACN) compared to NL129 (20% ACN) may indicate that the cross-linking reaction with the carbodiimide cross-linker has not fully progressed in K834 under these conditions. Differences in carboxylic acid functionality, polymer microstructure, and cross-linking kinetics between the latex grades can influence the accessibility of reactive sites and the efficiency of cross-link formation. At the higher curing temperature of 110 °C, the increased thermal energy likely promotes more complete cross-linking in K834, leading to a higher cross-link density and consequently lower swelling compared to NL129. This suggests that the swelling behavior is governed not only by ACN content but also by the extent of cross-link formation and curing temperature, which together determines the final network structure of the XNBR films.^17^

In XNBR systems with a high-ACN content, the increased polarity of the polymer chains leads to stronger intermolecular attractions and tighter molecular packing. This inherently restricts the mobility of the polymer chains and reduces the accessibility of reactive sites such as carboxylic acid groups (−COOH), which are crucial for cross-linking. As a result, traditional cross-linking methods may become less efficient under these conditions, particularly at lower curing temperatures, where the thermal energy is insufficient to overcome the reduced chain mobility. To address this challenge, it becomes essential to adopt cross-linking strategies that are both highly reactive and compatible with the high polarity of the XNBR matrix. Ionic and covalent cross-linking mechanisms can still be effective, but they must be tailored to the unique structural characteristics of high-ACN latex. Specifically, ionic cross-linking involving metal ions such as Zn^2+^ or covalent approaches using multifunctional AF cross-linkers can facilitate the formation of durable networks even at reduced thermal input. In this context, PCDI emerges as a promising AF cross-linker. It reacts efficiently with carboxyl groups to form stable covalent bonds, typically through the formation of urethane linkages. Due to its reactivity and compatibility with polar environments, PCDI is particularly suited to high-ACN XNBR systems, where it can achieve effective cross-linking at lower curing temperatures. This not only helps maintain the mechanical and barrier properties of the resulting latex films but also contributes to a more energy efficient and sustainable manufacturing process. Therefore, the use of PCDI in high-ACN XNBR formulations presents a viable solution for overcoming the limitations of traditional curing approaches, aligning with industry goals for both performance and environmental responsibility.^17,18^

Although the selected XNBR latex grades differ in both the ACN content and carboxylic acid monomer levels, these variations reflect the compositional differences commonly present in commercially available latex materials. The study therefore aims to evaluate the performance of accelerator-free cross-linking systems across different industrially relevant latex grades rather than isolating a single compositional variable. This approach provides a more practical understanding of how variations in polymer polarity and reactive carboxylic sites influence cross-link formation, mechanical performance, and aging behavior in AF-XNBR glove systems.^5^ It should be noted that the comparison between different latex grades in this study does not represent a single-variable system as the materials differ in multiple physicochemical characteristics, including ACN content, carboxylic acid functionality, molecular weight, and particle properties. Consequently, the observed performance differences are likely to be influenced by a combination of these factors. Nevertheless, this approach reflects industrial practice, where commercially available latex grades are evaluated on the basis of overall performance rather than isolated compositional parameters.

The swelling index exhibits an inverse relationship with cross-link density, where a lower swelling index corresponds to a more tightly cross-linked network as shown in Table 4. The reduced swelling observed in aged samples suggests an increase in cross-link density, likely due to postcuring effects or continued reaction between carbodiimide groups and residual carboxylic functionalities. Additionally, the consistently lower swelling index of NL117-based samples indicates a higher intrinsic cross-link density compared to other latex grades. Variations in curing temperature, however, result in comparatively smaller changes in both swelling index and calculated cross-link density. Overall, the Flory–Rehner analysis quantitatively supports the swelling results, providing strong evidence that aging and latex grade are the dominant factors governing the network structure in these systems.

**Table 4:** Results of Cross-Linked Density Properties

	cross-link density (×10^–5^ mol/cm^3^)	
latex gloves/cure temperature	unaged	aged
K834/65 °C	4.5	6.5
K834/90 °C	4.4	6.3
K834/110 °C	5.2	5.6
NL129/65 °C	5.3	7.2
NL129/90 °C	4.9	6.4
NL129/110 °C	4.7	6.6
NL117/65 °C	11.5	13.5
NL117/90 °C	10.8	12.7
NL117/110 °C	10.2	12.3

The calculated cross-link density, obtained via the Flory–Rehner equation, reveals a clear dependence on latex grade, with NL117 consistently exhibiting the highest values. This behavior can be attributed to differences in ACN content, as increasing ACN enhances the polarity of the polymer backbone, leading to stronger intermolecular interactions and reduced solvent uptake in nonpolar media such as toluene. As a result, samples with higher ACN contents exhibit lower swelling indices and correspondingly higher calculated cross-link densities. This is consistent with established findings that increased polarity and cohesive energy density in nitrile rubber systems restrict chain mobility and promote the formation of a tighter network structure.^19^

#### 4.1.3 Durability Properties

While 42 min is the target time for the durability of nitrile gloves, a maximum of 120 min is needed for this specific study in order to be fully comprehend the potential of the XNBR latex gloves. All unaged samples were successful for the durability test as listed in Table 5. Considerable aged samples at 70 °C for 7 days met the conditions as well.

**Table 5:** Results of Durability Properties^a^

	unaged sample		aged sample	
latex/cure temp.	crotch (min)	palm (min)	crotch (min)	palm (min)
K834/65 °C	120	120	43	69
K834/90 °C	120	120	55	72
K834/110 °C	120	120	68	98
NL129/65 °C	120	120	82	110
NL129/90 °C	120	120	90	120
NL129/110 °C	120	120	111	120
NL117/65 °C	120	120	115	120
NL117/90 °C	120	120	120	120
NL117/110 °C	117	120	120	120

a Thickness of gloves: 55 μm.

Table 6 emphasizes the durability of the performance. It reflects how well a glove maintains mechanical strength (especially in stress-prone regions such as the crotch) after accelerated aging, which simulates long-term use or exposure to heat, oxidation, and stress. Therefore, higher retention is equivalent to better durability, stronger cross-linked network, and greater resistance to degradation.^11^

**Table 6:** Results of Durability Retention Properties^a^

latex	temp (°C)	crotch retention (%)	palm retention (%)
K834	65	35.8	57.5
K834	90	45.8	60.0
K834	110	56.7	81.7
NL129	65	68.3	91.7
NL129	90	75.0	100.0
NL129	110	92.5	100.0
NL117	65	95.8	100.0
NL117	90	100.0	100.0
NL117	110	102.6	100.0

a Thickness of gloves: 55 μm.

K834 latex shows lower retention after aging (crotch retention: ∼ 36–57%), suggesting that it is less stable over time, particularly at lower curing temperatures. NL129 latex has moderate-to-high retention (68–93%), improving significantly with higher curing temperatures. NL117 latex performs the best, with retention values approaching or exceeding 100%. This indicates excellent aging resistance, even at lower curing temperatures (65 °C). Durability retention is crucial in glove applications, especially in industrial, medical, or chemical-handling settings where gloves must maintain performance over time or under stress, and aged degradation could pose safety risks.^17^ This result helps to identify the optimal latex and curing combination for longevity, justify the use of NL117 and high-temperature curing in high-performance applications, and support the effectiveness of the accelerator-free system in maintaining glove durability.

PCDI acts as an alternative cross-linker that forms covalent bonds with carboxylic acid groups in XNBR latex. Unlike sulfur-based systems, PCDI forms thermally stable urea-like linkages, which are more resistant to oxidation and thermal degradation. Cross-linking improves mechanical integrity as it aged and continues gradually over time (postcuring), particularly at lower temperatures like 65 °C. PCDI’s effectiveness is enhanced in latex with higher polarity (like NL117), as the cross-linking reaction with carboxylic acid groups is more efficient. Retention above 100% (as seen in NL117) suggests postcuring cross-linking may actually strengthen the glove over time, which is highly desirable for long-term durability.^18^

### 4.2 Effect of Thickness on AF-XNBR Glove Properties

#### 4.2.1 Modulus Strength Properties

The effect of thickness in XNBR gloves is evaluated from the results of modulus and tear strength that are particularly important, as they directly reflect the mechanical performance of the gloves. A constant curing temperature of 110 °C was used to allow easier comparison with commercial gloves, since this temperature is commonly applied in XNBR glove manufacturing. According to Figure 4, the bar graph shows the modulus at 100% and 300% elongation of XNBR gloves with different thicknesses (55, 70, and 90 μm) and latex grades (K834, NL129, and NL117), including commercial glove comparisons (Glove A, B, and C with thickness of 55, 70, and 90 μm, respectively).

**Figure 4:**
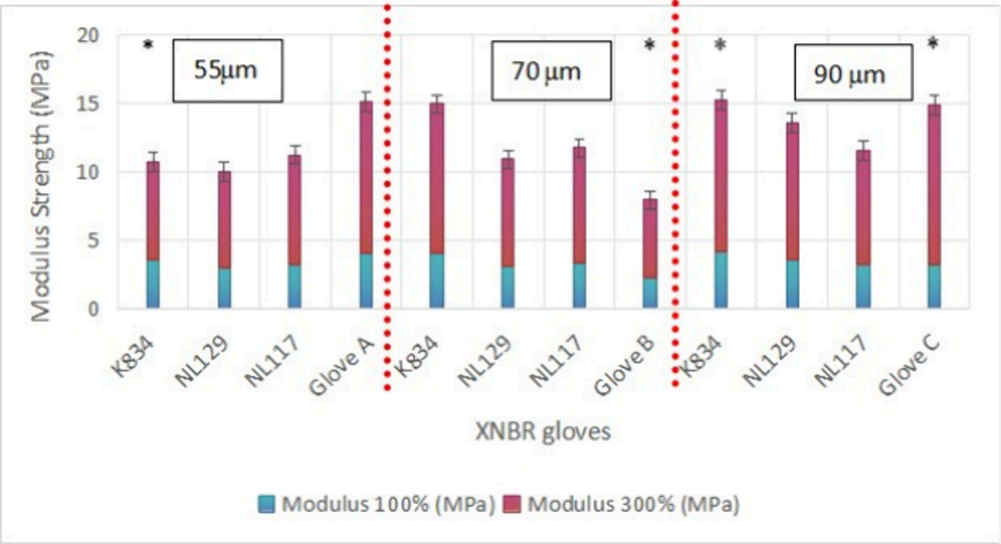
Modulus strength comparison of unaged XNBR gloves with commercial gloves (curing temperature: 110 °C) *p < 0.05.

All three XNBR grades (K834, NL129, and NL117) with a thickness of 55 μm show lower modulus values (both at 100% and 300%) compared to the commercial Glove A. This means the experimental gloves are more flexible or softer but may stretch more easily under load. Generally, lower modulus is equivalent to higher flexibility, which may be favorable for comfort and dexterity.^20^ Clearly, K834, despite lower modulus, still shows balanced stiffness between 100% and 300%, suggesting consistent stretch resistance. As thickness increases from 55 to 90 μm, both 100% and 300% modulus values generally increase. The thicker gloves (90 μm) show higher modulus, particularly in the K834 and Glove C samples. This implies better mechanical strength and resistance to deformation as thicker gloves require more force to stretch. This is expected because thicker films tend to have greater resistance to elongation, higher internal cohesion, and more material resisting the stretch. As modulus is thickness-dependent, the thickness of the glove tends to increase modulus values.^21^ Therefore, film thickness should always be taken into consideration when making direct mechanical comparisons.

XNBR gloves of K834, NL129, and NL117 show comparable performance, and these XNBR gloves show modulus values approaching commercial glove’s modulus values in the graph, indicating good mechanical performance from PCDI cross-linking. According to the findings, PCDI cross-linking helps to create tight, thermally stable networks and improve modulus and offers mechanical resistance that is on par with commercial gloves that have been sulfur-cured. For that reason, even with thinner gloves, proper cross-linking (via PCDI) can deliver comparable modulus performance, enabling lighter, more dexterous gloves without sacrificing mechanical strength.^22^

#### 4.2.2 Tear Strength Properties

Across all latex grades and thicknesses, the aged samples consistently exhibit higher tear strength than unaged ones as observed in Figure 5. For example, K834 at 70 μm increases from ∼65 kN/m (unaged) to ∼150 kN/m (aged). This suggests that thermal aging leads to additional cross-linking, strengthening the network, and improving crack resistance.^23^ PCDI may continue reacting postcure, forming more stable urethane linkages, which increase tear resistance over time. As film thickness increases from 55 to 90 μm, tear strength also increases, both in unaged and aged conditions. K834 at 90 μm shows the highest values (∼160 kN/m aged), reflecting stronger material integrity. This result indicates thicker films contain more polymer chains per unit area, offering greater resistance to tearing and more volume also accommodates more cross-linking sites which enhanced network strength.^24^

**Figure 5:**
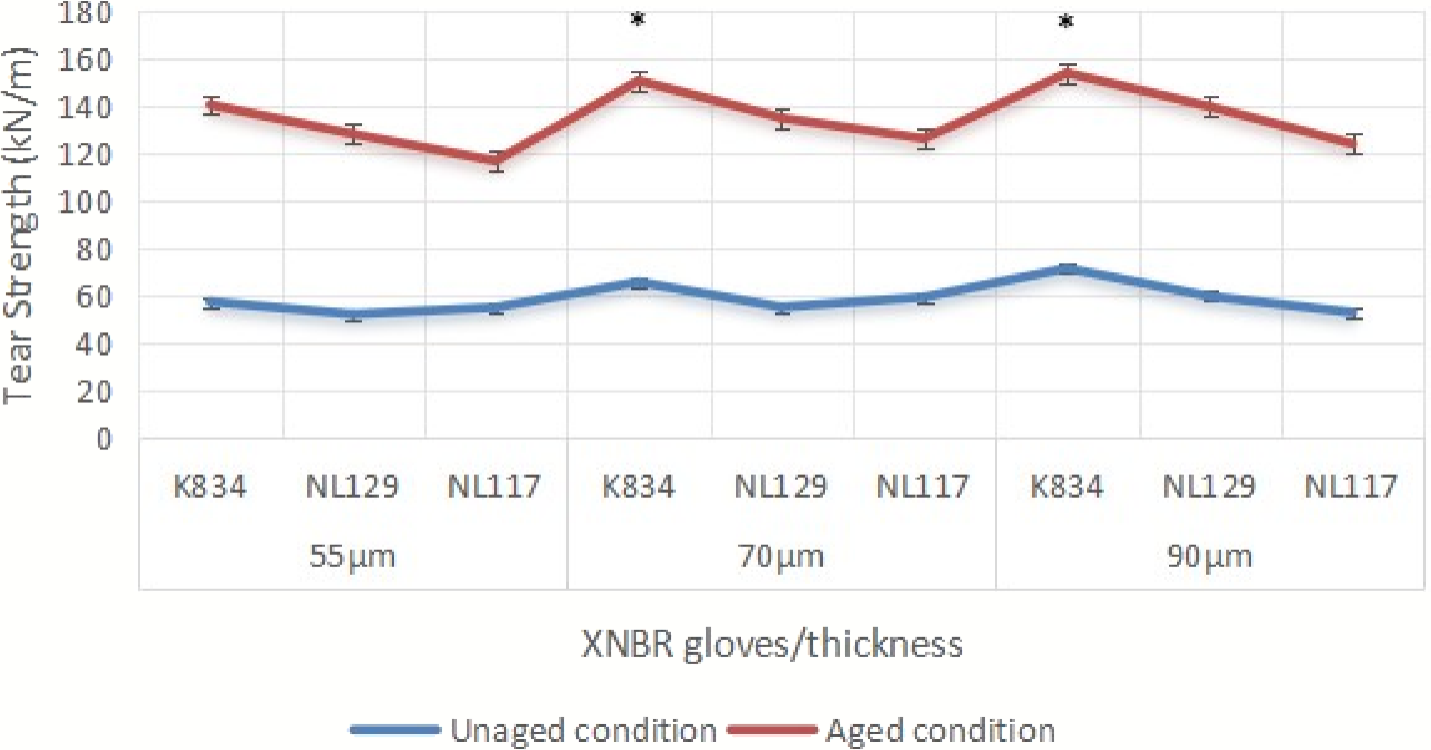
Tear strength of unaged and aged XNBR gloves (curing temperature: 110 °C) *p < 0.05.

Tear strength does not depend solely on glove thickness since it is largely influenced by how well the polymer chains are cross-linked. In Figure 5, 55 μm gloves still show acceptable tear strength, especially when aged. This suggests that the PCDI cross-linking system continues to enhance the internal network even in thin films. Thin gloves with a high-quality, uniform cross-linked network can resist tearing nearly as well as thicker ones.^25^ So, 55 μm gloves cured with PCDI still show tear strength comparable to thicker commercial gloves because of effective stress distribution.

In addition, uniform film formation during dipping and drying ensures that no weak points occur. Even at low thickness, if the glove surface is homogeneous, then the tear stress can be absorbed efficiently. Also, poorly processed thick gloves with internal voids or uneven cross-linking may perform worse than well-processed thin gloves.^25^ The graph shows a large jump in the tear strength after aging, especially in thin gloves. This implies that postcuring cross-linking continues to reinforce the structure over time, which is a big advantage of the PCDI system.

### 4.3 Effect of Curing Temperature on AF-XNBR Glove Properties

#### 4.3.1 Tensile Strength Properties

In order to reduce variables, a constant glove thickness of 55 μm was used to study the effect of curing temperature in XNBR gloves. The present industry requirement for thinner gloves led to the selection of this thickness. All samples were evaluated in accordance with the requirements of ASTM D6319. The bar graph in Figure 6 illustrates the tensile strength (in MPa) of three different XNBR latex grades, which are K834, NL129, and NL117 that were cured at 65, 90, and 110 °C. It shows the results of comparing the properties of XNBR gloves cross-linked at three different temperatures. All results are similar across the different temperatures. For example, the tensile strength of the latex films cured at 65 °C reached about 30 MPa, which is similar to 110 °C. In particular, K834 latex shows consistently high tensile strength across all curing temperatures (∼30 MPa). There is a slight improvement from 65 to 110 °C, showing good thermal response. It was best overall performance among the three latex types. As for NL129 latex, the result shows a decrease in tensile strength at 90 °C. However, the performance recovers slightly at 110 °C but still remains below 65 °C level. These suggests that midrange curing may negatively affect cross-linking quality for this formulation.^21^ Meanwhile, NL117 latex exhibits similar trend to NL129, with the highest strength at 65 °C. Even so, the strength decreases at 90 °C but shows modest recovery at 110 °C. This indicates that higher curing temperatures do not significantly enhance performance. Overall, K834 is the most thermally stable latex across all temperatures, maintaining superior mechanical strength. NL129 and NL117 are more temperature-sensitive, possibly due to formulation differences or cross-linking efficiency. The drop at 90 °C for NL129 and NL117 may suggest suboptimal cross-linking or premature degradation at this temperature.^5^

**Figure 6:**
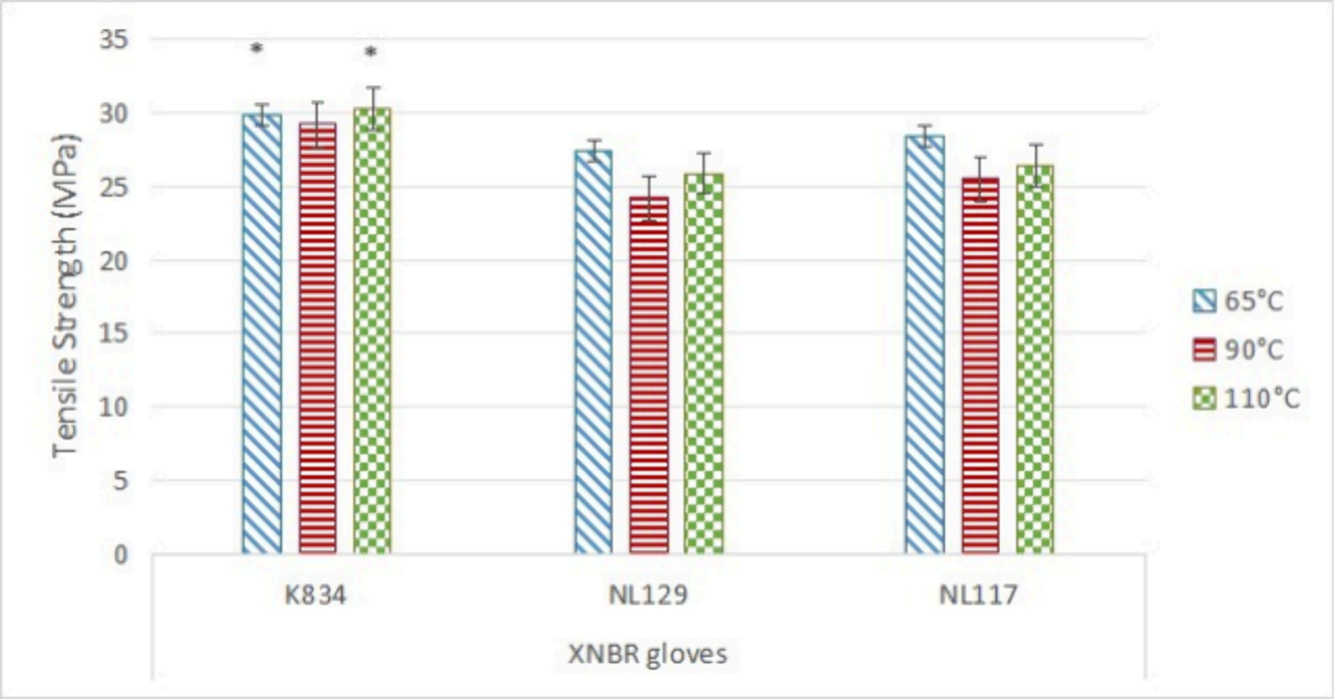
Tensile strength of XNBR gloves with three different curing temperatures (thickness of gloves: 55 μm) *p < 0.05.

Tensile strength is a direct indicator of cross-link density and molecular integrity in the rubber network. Typically, higher curing temperatures (e.g., 110 °C) promote more complete and faster cross-linking, resulting in stronger, more elastic films. Yet, the results show comparable or even superior tensile strength at low curing temperature (65 °C), suggesting effective cross-linking even at mild conditions, likely due to the role of PCDI. The key features of PCDI include low-temperature reactivity, which is unlike sulfur-based systems, PCDI can initiate cross-linking at temperatures as low as 65 °C. PCDI may continue forming cross-links even after the initial cure, leading to comparable strength at low and high temperatures, and the bonds formed are resistant to degradation during heat aging. Hence, PCDI likely enabled early stage network formation at 65 °C, producing films with high tensile strength without needing high thermal energy.^18^

Figure 7 demonstrates all XNBR latex gloves, regardless of latex grade (K834, NL129, or NL117) and curing temperature (65, 90, or 110 °C), demonstrated elongation at break values exceeding 500%. Statistical analysis using an independent *t* test confirmed that there were no significant differences (*p* > 0.05) in elongation at break among the different latex grades and curing temperatures. This indicates that the polymer network formed was highly elastic and well cross-linked across all conditions, maintaining superior flexibility without compromising the structural cohesion of the films. The indication of flexibility and elasticity is because XNBR is known for combining good tensile strength with high flexibility due to its elastomeric nature. The presence of carboxylic groups enhances compatibility and cross-linking potential without significantly compromising chain mobility, allowing for high stretchability.^26^ All three latex grades likely formed adequate cross-link networks at each curing temperature, sufficient to retain elasticity while maintaining structural integrity. Even at the lowest curing temperature (65 °C), the curing was likely effective enough to develop a network that supports elongation beyond 500%.

**Figure 7:**
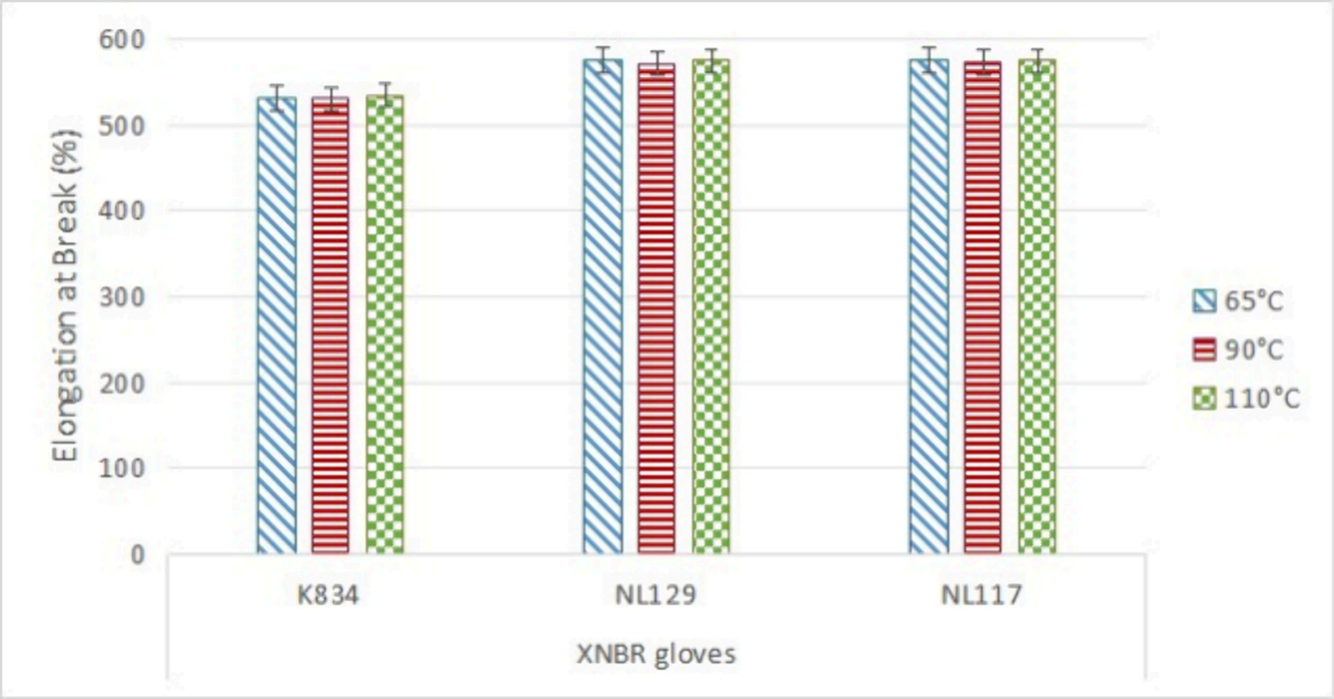
Elongation at break of XNBR gloves with three different curing temperature (thickness of gloves: 55 μm).

Basically, using PCDI as an AF cross-linker for XNBR gloves at different curing temperatures showed no significant differences in mechanical properties. PCDI effectively produced high-quality XNBR gloves even at a low curing temperature of 65 °C. This is due to the high reactivity of the carbodiimide group with carboxylate, enabling cross-linking at lower temperatures. While the carbodiimide group can also react with water, the micelle structure of PCDI prevents this, allowing efficient cross-linking of XNBR.^18^

The effectiveness of PCDI at low curing temperature in accordance with ASTM573, 2016, a standard procedure for evaluating the degradation of rubber products in an air oven, an aging research, was carried out to highlight an intriguing aspect of PCDI cross-linking of gloves at 70 °C for 7 days. The graphs in Figures 8 and 9 demonstrate that XNBR gloves with a constant thickness of 55 μm cured at 65 °C using a PCDI cross-linking system retain excellent mechanical properties even after undergoing aging processes. Statistical analysis (*t* test) confirmed that aging resulted in a significant increase in tensile strength (*p* < 0.05) for all latex grades, while no significant differences (*p* > 0.05) were observed between the different latex grades within the same aging condition. This is a significant result, especially considering that low-temperature curing typically leads to weaker cross-linking in conventional sulfur-based systems. Despite being cured at just 65 °C, the gloves maintain strong tensile strength and elongation at break after aging. This suggests that PCDI forms stable chemical bonds, particularly through the reaction between carbodiimide groups and carboxylic acids in XNBR, which are less prone to degradation under thermal or oxidative stress compared to sulfur cross-links. The consistency in performance across different latex grades (K834, NL129, and NL117) highlights the versatility of PCDI as a cross-linking agent. Regardless of the latex composition, the aged gloves show minimal reduction or even improvement in performance, implying that PCDI enables uniform cross-linking efficiency across varying XNBR gloves. The ability to cure effectively at 65 °C brings substantial energy savings and process flexibility in glove manufacturing. It also supports AF glove production, reducing the risk of allergic reactions caused by residual chemical accelerators (e.g., thiurams or carbamates).^11^ Similar studies and results can be found in ref 27, where this study reports that virgin XNBR films exhibit an elongation at break of approximately 530%. The high elasticity is attributed to the inherent properties of XNBR. Also, ref 28 explains that in this research, it was observed that increasing the XNBR content in the composite led to a significant improvement in the elongation at break, with enhancements up to 46%. This indicates the role of XNBR in enhancing the flexibility of the material.

**Figure 8:**
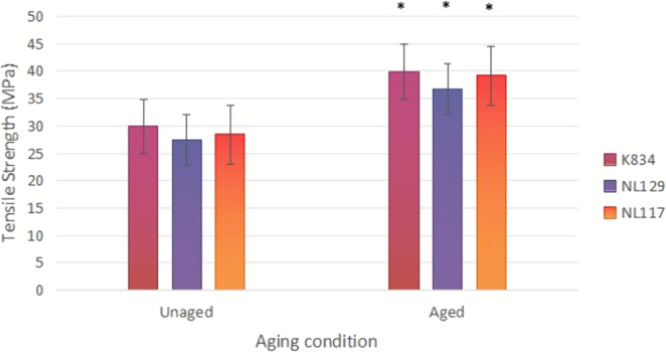
Tensile strength of unaged and aged XNBR gloves (thickness of gloves: 55 μm, curing temperature: 65 °C) *p < 0.05.

**Figure 9:**
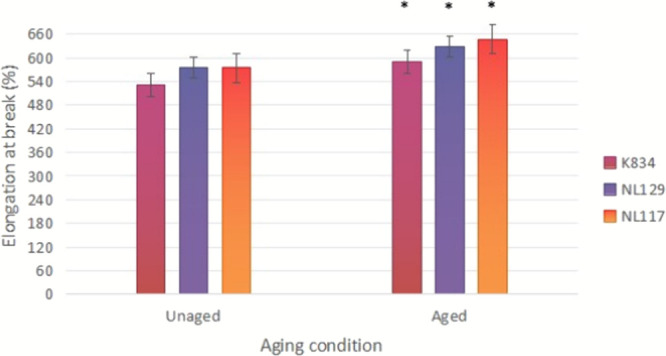
Elongation at break of unaged and aged XNBR gloves (thickness of gloves: 55 μm, curing temperature: 65 °C) *p < 0.05

Aging simulates long-term usage or storage conditions and tests the glove’s resistance to oxidative and thermal degradation. Heat aging normally results in loss of elasticity or tensile strength, softening or hardening (based on formulation), and chain scission (weakening). However, in this study the PCDI-cured XNBR gloves still retain excellent mechanical properties. This suggests the cross-links remain intact under aging conditions due to the thermal stability of PCDI-derived bonds. Also, postcuring cross-linking may even occur during aging, strengthening the polymer network further (especially if residual unreacted PCDI is present). Additionally, the carboxylated structure of XNBR also contributes to polar–polar interactions, adding to durability.^29^

Using lower curing temperatures in glove production contributes significantly to several United Nations Sustainable Development Goals (SDGs). First, it supports SDG 7 (Affordable and Clean Energy) by reducing energy consumption during the curing process. This improves overall energy efficiency in industrial operations, aligning with the global goal of promoting cleaner energy use.^27^ Second, it aligns with SDG 12 (Responsible Consumption and Production). By optimizing curing conditions, manufacturers can reduce the use of resources, such as electricity and heat. This promotes more sustainable manufacturing practices and lowers the overall environmental impact of the production. Lastly, it contributes to SDG 13 (Climate Action: Reduced) where energy usage leads to lower greenhouse gas emissions, particularly when fossil fuels are the primary energy sources. This helps decrease the carbon footprint of glove production and supports efforts to combat climate change.^30^

As for Environmental, Social, and Governance (ESG) impact, lower curing temperatures in glove production contribute significantly to the reduction of CO_2_ emissions. Research has shown that energy-efficient curing strategies such as cross-linking XNBR latex with multifunctional epoxides not only maintain glove performance but can also reduce thermal load by up to 30%. Studies from institutions such as Montanuniversität Leoben and industry sources like Ruico Global support these findings, highlighting the potential of such innovations to improve environmental outcomes in manufacturing.^18^ Energy savings achieved through lower curing temperatures can result in cost reductions. These savings can be redirected toward enhancing employee welfare and investing in community development initiatives. Furthermore, the shift to produce gloves with reduced chemical accelerators improves workplace safety by lowering the risk of harmful chemical exposure for workers, thereby promoting a healthier and safer working environment. Implementing sustainable manufacturing practices demonstrates a company’s commitment to responsible governance. By adopting energy-efficient processes and reducing environmental impacts, companies can strengthen stakeholder confidence and ensure alignment with global ESG and sustainability reporting standards. This positions the organization as forward-thinking and transparent in its operational strategies.^31^

## 5 Conclusions

This study confirms that accelerator-free cross-linking using polycarbodiimide (PCDI) produces XNBR gloves with durability, swelling resistance, and aging performance comparable to or better than accelerator-cured systems. Stable tensile strength and elongation at break across curing temperatures and latex grades indicate robust and elastic networks. Elongation at break exceeding 500% supports suitability for high-flexibility applications. Latex-grade selection enables performance tuning: K834 provides high flexibility, NL117 offers enhanced chemical resistance, and NL129 delivers a balanced combination of flexibility and oil resistance. Effective curing at lower temperatures broadens processing flexibility and reduces the energy demand. Overall, these results establish PCDI as a green and efficient cross-linker for accelerator-free XNBR gloves, supporting high-performance, low-allergen products for medical, cleanroom, and industrial applications.

## Supplementary Material



## Notes

Additional experimental details on durability test machine, supplementary figures and tables, variation of FTIR spectra of XNBR-PCDI film with different latex grades, swelling index analysis data of unaged and aged XNBR gloves, modulus strength comparison of unaged XNBR gloves with commercial gloves, tear strength of unaged and aged XNBR gloves, tensile strength and elongation at break of XNBR gloves with three different curing temperatures, tensile strength and elongation at break of unaged and aged XNBR gloves (thickness of gloves: 55 μm, curing temperature: 65 °C), general effect of acrylonitrile (ACN) content on XNBR properties based on literature reports, formulation of XNBR cross-link system, experimental designs, results of cross-link density properties, results of durability properties, and results of durability retention properties (PDF)

## References

[1] Qu S., Hao Y., Xiao Y., Xue J., Sui Z., Pan Y., Liang J., Zhu D., Wang C., Bian H. (2022). Reinforcing
mechanism and experimental study of environmentally friendly potassium
oleate in silica/natural rubber system. J. Appl.
Polym. Sci..

[2] Phalen R. N., Wong W. K. (2011). Integrity of Disposable
Nitrile Exam Gloves Exposed
to Simulated Movement. J. Occup Environ. Hyg.

[3] Hesselmans L.C. J., Derksen A. J., van den Goorbergh J.
A. M. (2006). Polycarbodiimide
crosslinkers. Prog. Org. Coat..

[4] Paran S. M., Naderi G., Mosallanezhad H., Movahedifar E., Formela K., Saeb M. R. (2020). Microstructure and
mechanical properties
of Carboxylated nitrile butadiene rubber/epoxy/XNBRgrafted halloysite
nanotubes nanocomposites. Polymers.

[5] Fleischmann D. D., Ayalur-Karunakaran S., Arbeiter F. (2018). Influence of crosslinker
and water on mechanical properties of carboxylated nitrile butadiene
rubber (XNBR). Polym. Test..

[6] Wells R., Hunt S., Hurley K., Rosati P. (2010). Laboratory assessment
of the effect of heavy rubber glove thickness and sizing on effort,
performance and comfort. Int. J. Ind.Erg..

[7] Nelson J. B., Mital A. (2007). An ergonomic evaluation
of dexterity and tactility with increase
in examination/surgical glove thickness. Ergonomics.

[8] Tang L., Wang S., Xiaoren X. L. V., Enqiu H. E., Yao H. (2017). Effect of
Curing Temperature on Mechanical and Tribological Properties of Nitrile-butadiene
Rubber. Lubr. Eng..

[9] Wan
Azman N., Fujiwara M., Enomoto N., Rashid A. A. (2024). Revealing
the unique characteristics and strength of polycarbodiimide as an
accelerator-free crosslinker for low-temperature processing of carboxylated
nitrile butadiene rubber gloves for hand protection. J. Vinyl Addit Technol..

[10] Khankishiyeva R., Mammadov A., Akhundzada H. V., Mammadova G., Khudaverdiev V., Azizova A., Salehov A., Valiyeva S., Moshiul Alam A. K. M. (2026). Enhanced chemical resistance and
mechanical properties
of HNBR elastomers via ethylphenylsilylurea (EPSU) based crosslinking
modification. Polym. Bull..

[11] Liu S., Jing Y., Tu J., Zou H., Yong Z., Liu G. (2022). Systematic investigation on the swelling behaviors of acrylonitrile-butadiene
rubber via solubility parameter and Flory-Huggins interaction parameter. J. Appl. Polym. Sci..

[12] Wang L., Ni Y., Qi X., Zhang L., Yue D. (2021). Synthesis of Low Temperature
Resistant Hydrogenated Nitrile Rubber Based on Esterification Reaction. Polymers.

[13] Sreenath P. R., Singh S., Satyanarayana M. S., Das P., Kumar K. D. (2017). Carbon
dot e Unique reinforcing filler for polymer with special reference
to physico-mechanical properties. Polymer.

[14] Wajge S. W., Das C. (2024). An Alternate Approach
of Cross-Linking XNBR via Ferric–Carboxylate
Interaction Assisted by Dimethylaminopyridine. ACS Appl. Polym. Mater..

[15] Tan Y., Zhang J., Wang Y. (2018). Chemical resistance and swelling
behavior of acrylonitrile-butadiene rubber composites in aggressive
solvents. J. Polym. Res..

[16] Wang H., Liu S., Liu G. (2023). Investigation on the
thermo-oxidative aging resistance
of nitrile-butadiene rubber/polyamide elastomer blend and the swelling
behaviors in fuels predicted via Hansen solubility parameter method. Polym. Degrad. Stab..

[17] Ogawa T., Shibasaki J., Wan Azman N. (2024). Development of low dermatitis
potential NBR gloves by accelerator-free crosslinking using polycarbodiimide
compounds. Mater. Today Commun..

[18] Grabmayer T., Manhart J. C., Kaiser S., Schaller R., Holzner A., Schlögl S. (2023). Crosslinking of XNBR latex with multifunctional
epoxides:
An energy-efficient curing strategy for the fabrication of accelerator-free
rubber gloves. J. Appl. Polym. Sci..

[19] Xin H., Shepherd D. E. T., Dearn K. D. (2013). Strength of poly-ether-ether-ketone:
Effects of sterilisation and thermal ageing. Polym. Test..

[20] Przybysz M., Marć M., Klein M., Saeb M. R., Formela K. (2018). Structural,
mechanical and thermal behavior assessments of PCL/PHB blends reactively
compatibilized with organic peroxides. Polym.
Test..

[21] Ammineni S. P., Nagaraju C., Lingaraju D. (2022). Thermal degradation of naturally
aged NBR with time and temperature. Mater. Res.
Express.

[22] Wan
Azman N., Abdullah N. A., Enomoto N., Rashid A. A. (2024). Effect
of Post-Processing Parameters of Accelerator-Free Crosslinker (AF)
on Mechanical Properties of Carboxylated Nitrile Butadiene Rubber
(XNBR) Latex Films. J. Phys.: Conf. Series.

[23] Kabe T., Matsumoto K., Terai S., Hikima T., Takata M., Miyake M., Taguchi S., Iwata T. (2016). Co-crystallization
phenomena in biosynthesized isotactic poly­[(R)-lactate-co-(R)-2-hydroxybutyrate]­s
with various lactate unit ratios. Polym. Degrad.
Stab..

[24] Loksupapaiboon K., Suvanjumrat C. (2024). Curing analysis of rubber film on a Hand-Shaped former
in the manufacturing of rubber gloves through novel OpenFOAM solver. International Journal of Heat and Fluid Flow.

[25] Guo Y. Y., Thing C. T., Chung L. L., Dinh-Toi C., Chiaki O., Pau L. S. (2019). Emerging crosslinking
techniques for glove manufacturers
with improved nitrile glove properties and reduced allergic risks. Mater. Today Commun..

[26] Lim H. M., Tan K. S. (2022). Carboxylated acrylonitrile
butadiene-natural rubber
latex blends with methyl methacrylate grafted natural rubber latex:
mechanical properties and morphology. Journal
of Rubber Research.

[27] Glove, Eco Innovative Technologies in Disposable Glove Manufacturing. Published March 3, 2025. Accessed May 24, 2025. https://ecogloves.co/blogs/resources/innovative-technologies-in-disposable-glove-manufacturing.

[28] Saberian M., Ghasemi F. A., Ghasemi I., Daneshpayeh S. (2019). Investigation
on tensile properties of epoxy/graphene nano-platelets/carboxylated
nitrile butadiene rubber ternary nanocomposites using response surface
methodology. Nanomaterials and Nanotechnology.

[29] Krzeminska S. M., Smejda-Krzewicka A. A., Leniart A., Lipinska L., Woluntarski M. (2020). Effects of
curing agents and modified graphene oxide on the properties of XNBR
composites. Polym. Test..

[30] Ruico Global Carboxylated Nitrile Latex: Revolutionizing Medical Examination Gloves with Superior Elasticity. Published May 19, 2025. Accessed May 24, 2025. https://www.ruicoglobal.com/news/carboxylated-nitrile-latex-revolutionizing-medical-examination-gloves-with-superior-elasticity.html?.

[31] Koziol, K. New modular manufacturing process for latex rubber gloves on course for net zero. Published November 26, 2021. Accessed May 24, 2025. https://blogs.cranfield.ac.uk/manufacturing/new-modular-manufacturing-process-for-latex-rubber-gloves-on-course-for-net-zero/?.

