# Supplementary material for: Physicomechanical Performance and Aging Behavior of Polycarbodiimide-Cross-Linked Accelerator-Free Carboxylated Nitrile Butadiene Rubber Latex Gloves
Source: ACS Omega. 2026 May 25;11(22):33005–15. doi: 10.1021/acsomega.6c02669 (PMC13261471; doi:10.1021/acsomega.6c02669)
Supplement: Supplementary file 1 [file ao6c02669_si_001.pdf]

Supporting Information

Physico-Mechanical Performance and Aging Behaviour of Polycarbodiimide-Crosslinked Accelerator-Free XNBR Latex Gloves

Nadia Wan Azman[a], Ryota Kamei[b], Toshikazu Matsuoka[b], Keisuke Ikeda[b], Yugo Kubono[b], Azura A. Rashid[a*]

[a]*School of Materials and Mineral Resources Engineering, Universiti Sains Malaysia, Engineering Campus, 14300 Nibong Tebal, Pulau Pinang, Malaysia*

[b]*Personal Protective Equipment Technology Dept. Midori Anzen Co., LTD., 5-27-1 Inari, Souka, Saitama 340-0003, Japan.*

FIGURES SI

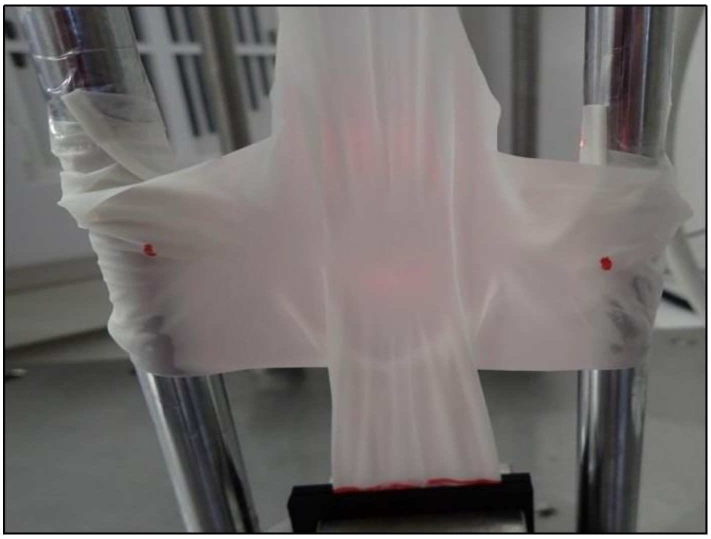

Figure S1 : Attachment of sample on the durability test machine

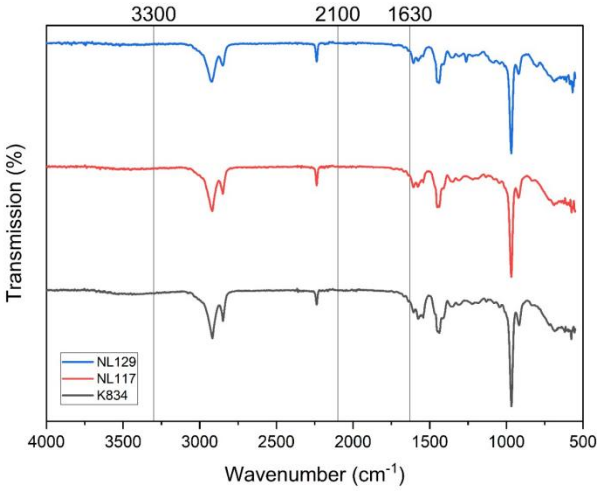

Figure S2 : Variation of infrared spectra of XNBR-PCDI film with different latex grades

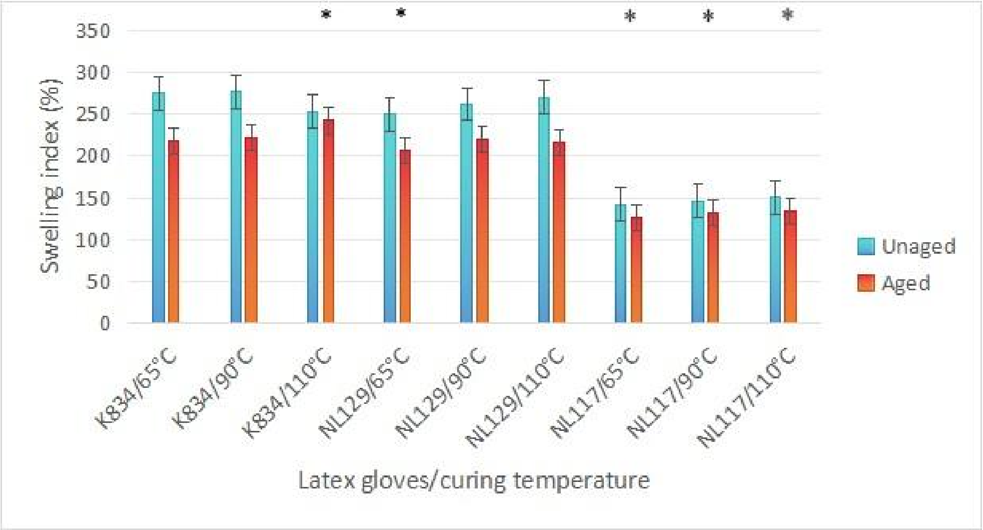

Figure S3 : Toluene swelling index of unaged and aged XNBR gloves
*Thickness of gloves: 55 µm
*$p < 0.05$

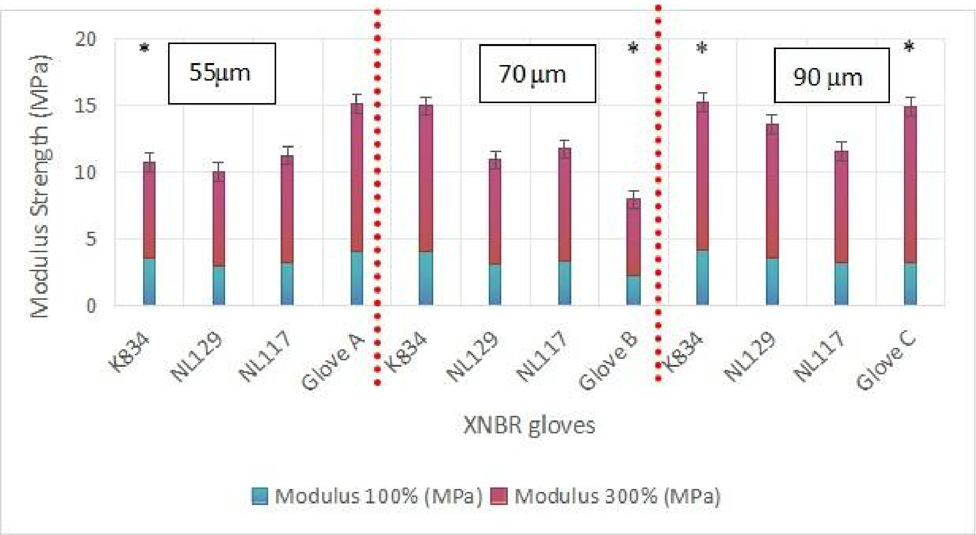

Figure S4 : Modulus strength comparison of unaged XNBR gloves with commercial gloves
(Curing temperature: 110°C)
*$p < 0.05$

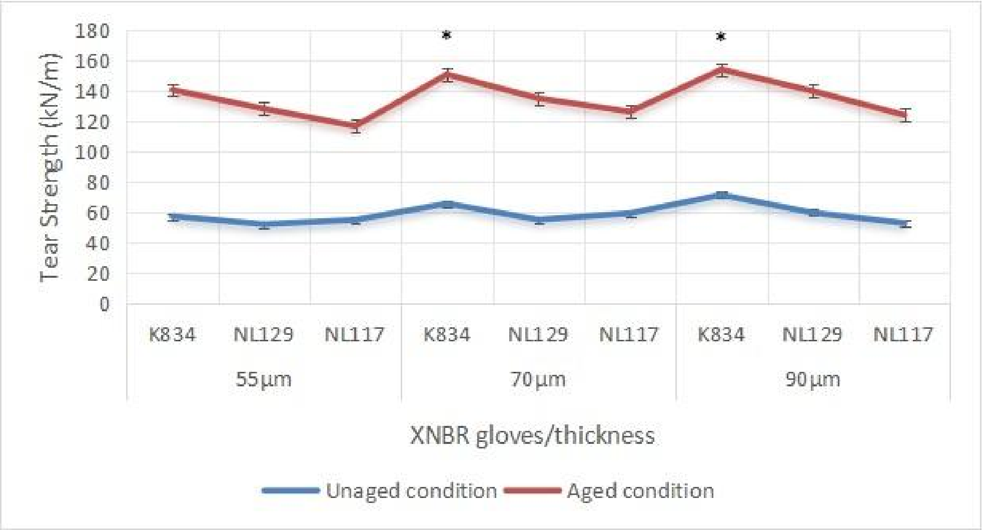

Figure S5 : Tear strength of unaged and aged XNBR gloves
(Curing temperature: 110°C)
*$p < 0.05$

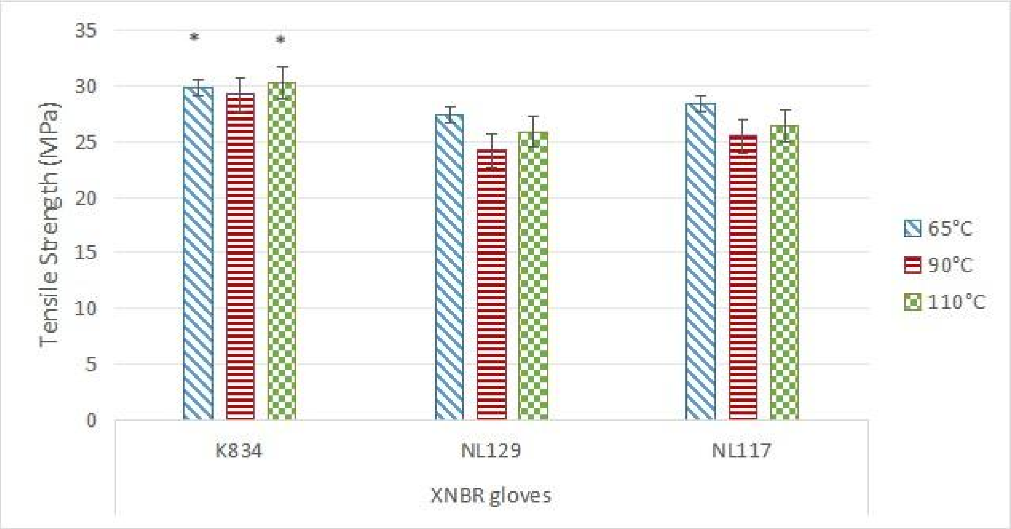

Figure S6 : Tensile strength of XNBR gloves with three different curing temperature
(Thickness of gloves: 55 µm)
*$p < 0.05$

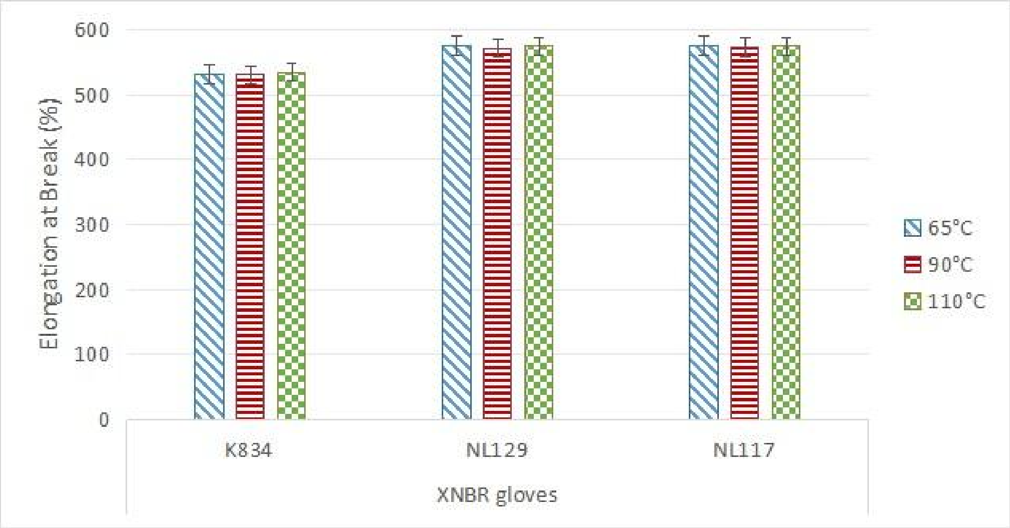

Figure S7 : Elongation at break of XNBR gloves with three different curing temperature
(Thickness of gloves: 55 µm)

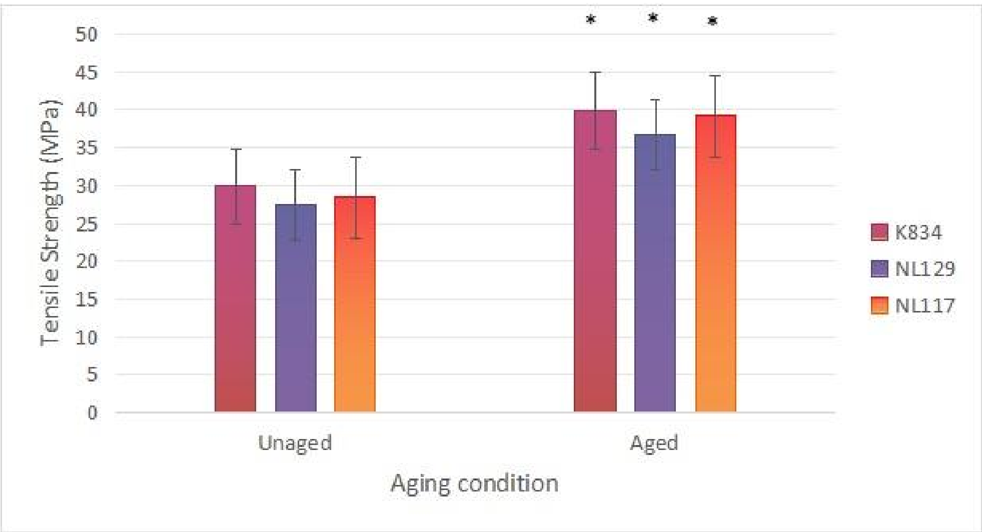

Figure S8 : Tensile strength of unaged and aged XNBR gloves
(Thickness of gloves: 55 µm, Curing temperature: 65°C)
*$p < 0.05$

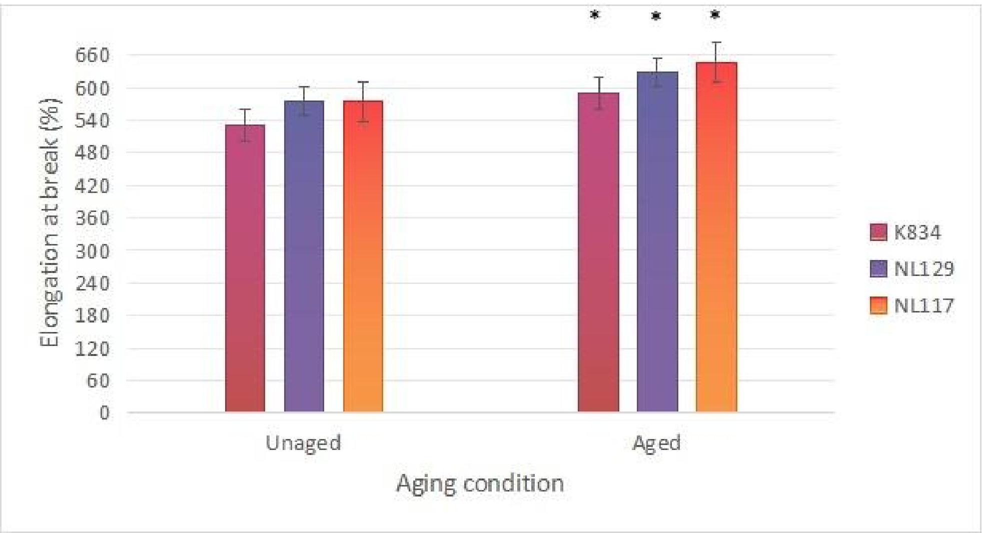

Figure S9 : Elongation at break of unaged and aged XNBR gloves
(Thickness of gloves: 55 µm, Curing temperature: 65°C)
*$p < 0.05$

TABLES SI

Table S1
General effect of acrylonitrile (ACN) content on XNBR properties based on literature reports.

ACN Content	Characteristics	Use Cases
Low (18–25%)	• Higher flexibility and softness<br>• Lower oil and chemical resistance<br>• Better elongation at break	Gloves requiring comfort and stretch, like examination or medical gloves
High (35–45%)	• Superior oil, fuel, and chemical resistance<br>• Lower elasticity • Stiffer feel	Industrial gloves for chemical handling, automotive, or oil & gas applications

*Source: Summarized from literature on nitrile rubber structure–property relationships.

Table S2
Formulation of XNBR crosslink system

Component	45% XNBR	5% KOH	50% ZnO	50% Antioxidant	70% TiO_2	40% PCDI
phr	100	2	1	0.2	2	0.5

[a] phr indicates part per hundred parts of rubber

Table S3
Experimental designs

Latex grade	K834	K834	K834	NL129	NL129	NL129	NL117	NL117	NL117
Curing Temperature (°C)	65	90	110	65	90	110	65	90	110

Table S4
Results of crosslink density properties

Latex gloves/Cure Temperature	Crosslink density ($\times 10^{-5}$ mol/cm^3)	
	Unaged	Aged
K834/65°C	4.5	6.5
K834/90°C	4.4	6.3
K834/110°C	5.2	5.6
NL129/65°C	5.3	7.2
NL129/90°C	4.9	6.4
NL129/110°C	4.7	6.6
NL117/65°C	11.5	13.5
NL117/90°C	10.8	12.7
NL117/110°C	10.2	12.3

Table S5
Results of durability properties

Latex/Cure Temp.	Unaged sample		Aged sample	
	Crotch (min)	Palm (min)	Crotch (min)	Palm (min)
K834/65°C	120	120	43	69
K834/90°C	120	120	55	72
K834/110°C	120	120	68	98
NL129/65°C	120	120	82	110
NL129/90°C	120	120	90	120
NL129/110°C	120	120	111	120
NL117/65°C	120	120	115	120
NL117/90°C	120	120	120	120
NL117/110°C	117	120	120	120

*Thickness of gloves: 55 µm

Table S6
Results of durability retention properties

Latex	Temp (°C)	Crotch Retention (%)	Palm Retention (%)
K834	65	35.8	57.5
K834	90	45.8	60.0
K834	110	56.7	81.7
NL129	65	68.3	91.7
NL129	90	75.0	100.0
NL129	110	92.5	100.0
NL117	65	95.8	100.0
NL117	90	100.0	100.0
NL117	110	102.6	100.0

*Thickness of gloves: 55 µm
